# Bipartite Activation of Sensory Neurons by a TRPA1 Agonist Allyl Isothiocyanate Is Reflected by Complex Ca^2+^ Influx and CGRP Release Patterns: Enhancement by NGF and Inhibition with VAMP and SNAP-25 Cleaving Botulinum Neurotoxins

**DOI:** 10.3390/ijms24021338

**Published:** 2023-01-10

**Authors:** Mariia Belinskaia, Jiafu Wang, Seshu Kumar Kaza, Caren Antoniazzi, Tomas Zurawski, J. Oliver Dolly, Gary W. Lawrence

**Affiliations:** International Centre for Neurotherapeutics, Dublin City University, Collins Avenue, D09 V209 Dublin, Ireland

**Keywords:** AITC, botulinum neurotoxins, calcitonin gene-related peptide, trigeminal ganglion neurons, nerve growth factor, SNAP-25, TRPA1, TRPV1, VAMP/synaptobrevin

## Abstract

The trafficking of transient receptor potential (TRP) channels to the plasma membrane and the release of calcitonin gene-related peptide (CGRP) from trigeminal ganglion neurons (TGNs) are implicated in some aspects of chronic migraines. These exocytotic processes are inhibited by cleavage of SNAREs with botulinum neurotoxins (BoNTs); moreover, type A toxin (/A) clinically reduces the frequency and severity of migraine attacks but not in all patients for unknown reasons. Herein, neonatal rat TGNs were stimulated with allyl isothiocyanate (AITC), a TRPA1 agonist, and dose relationships were established to link the resultant exocytosis of CGRP with Ca^2+^ influx. The CGRP release, quantified by ELISA, was best fit by a two-site model (EC_50_ of 6 and 93 µM) that correlates with elevations in intracellular Ca^2+^ [Ca^2+^]_i_ revealed by time-lapse confocal microscopy of fluo-4-acetoxymethyl ester (Fluo-4 AM) loaded cells. These signals were all blocked by two TRPA1 antagonists, HC-030031 and A967079. At low [AITC], [Ca^2+^]_i_ was limited because of desensitisation to the agonist but rose for concentrations > 0.1 mM due to a deduced non-desensitising second phase of Ca^2+^ influx. A recombinant BoNT chimera (/DA), which cleaves VAMP1/2/3, inhibited AITC-elicited CGRP release to a greater extent than SNAP-25-cleaving BoNT/A. /DA also proved more efficacious against CGRP efflux evoked by a TRPV1 agonist, capsaicin. Nerve growth factor (NGF), a pain-inducing sensitiser of TGNs, enhanced the CGRP exocytosis induced by low [AITC] only. Both toxins blocked NGF-induced neuropeptide secretion and its enhancement of the response to AITC. In conclusion, NGF sensitisation of sensory neurons involves TRPA1, elevated Ca^2+^ influx, and CGRP exocytosis, mediated by VAMP1/2/3 and SNAP-25 which can be attenuated by the BoNTs.

## 1. Introduction

Affecting >14% of the population, migraines are ranked the sixth most prevalent and disabling disorder worldwide, being even more common among young adults and women [1,2]. It is characterised by moderate to severe headaches accompanied by nausea, vomiting, photophobia, phonophobia, cognitive impairment, fatigue, and mechanical allodynia [3]. Clinical studies identified a correlation between acute migraine headaches [4] and increased levels in the cranial blood of the pain-mediating calcitonin gene-related peptide (CGRP). It is a 37 amino acid neuropeptide expressed highly in small to mid-sized trigeminal ganglion neurons (TGNs), especially within unmyelinated C-fibres that innervate the craniofacial region and is released by Ca^2+^-regulated exocytosis. CGRP acts on a G-protein coupled receptor, composed of the calcitonin receptor-like receptor and receptor activity modifying protein isoform 1 [3]. These are expressed widely in the central nervous system and peripheral vascular smooth muscle but are also present on large TGNs plus Aδ fibres that do not express CGRP [5] and may occur on satellite glial cells [6]. CGRP is a potent vasodilator [5], an instigator of neurogenic inflammation [7], and transmits nociceptive signals from trigeminal nociceptors to neurons that project centrally [5]. It might also sensitise TGNs indirectly by the stimulation of nitric oxide release from satellite glial cells [6]. Infusion of CGRP triggers migraine-like attacks in migraineurs but only mild headache symptoms in healthy patients [8,9]. Thus, CGRP signaling is a verified target for migraine therapy, with monoclonal antibodies that neutralise this neuropeptide or its receptor, and gepants that inhibit its binding to receptors, all being used clinically [3,10].

Transient receptor potential (TRP) channels can occur on CGRP-containing TGNs, and when activated, permit the influx of cations including Ca^2+^, a stimulant of CGRP release. They are found less extensively on non-peptidergic sensory neurons [10] and non-neuronal cells. Although several TRP channels are being actively investigated for potential roles in migraine, in view of their expression on trigeminal nociceptors and responsiveness to exogenous factors associated with increased headache risk [10,11], particular attention has been focused on TRP subfamily ankyrin member 1 (TRPA1). This is due to its broad sensitivity to a wide range of chemicals that are initiators of, or sensitisers for, migraines, including reactive nitrogen and oxygen species, prostaglandins, chlorine, formaldehyde, cigarette smoke, acrolein, and headache-inducing plant extracts such as mustard oil and umbellulone. On the other hand, herbal extracts and pharmaceuticals efficacious for treating migraines have been shown to antagonise TRPA1 [7,11]. Allyl isothiocyanate (AITC, from mustard oil) activates TRPA1 by chemical reaction with nucleophilic cysteine (Cys) and lysine (Lys) residues [12,13]. Molecular details on the activation of TRPA1 by AITC and related electrophiles are being resolved by a combination of chemical labelling, mutagenesis, and structure-function studies (see Section 3). TRPA1 activation by isothiocyanates is followed by strong desensitisation [14,15,16]; self- and cross-desensitisation of TRPA1 and TRP subfamily vanilloid member 1 (TRPV1), seems to be relevant to the anti-migraine properties of some analgesics [17].

Nerve growth factor (NGF) induces pain in animals [18] and lowers pain thresholds in humans [19]; its levels have been shown to be elevated in chronic migraine patients [20,21] but not in all studies [22]. NGF sensitises sensory neurons in vitro by inducing the transfer of the capsaicin (CAP) receptor TRPV1 from intracellular reserves to the cell surface and, to a lesser extent, by modulation of channel activity [23]. Another algesic, tumour necrosis factor-alpha (TNFα), is elevated in patients’ serum during episodic migraine attacks [24] and is persistently higher in the cerebrospinal fluid of chronic migraineurs [25]. Similar to NGF, TNFα mobilises intracellular stores of TRPV1 to the plasma membrane of TGNs in vitro [26,27]. Accordingly, pre-treating sensory neurons with TNFα or NGF enhances the currents elicited by CAP (a TRPV1 activator), intensifies the resultant increases of intracellular Ca^2+^ concentration ([Ca^2+^]_i_), as well as augmenting CAP-evoked CGRP release [26,27,28]. The surface transfer of TRPV1 can be inhibited by pre-treatment of the TGNs with botulinum neurotoxin type A (BoNT/A) [26], a bacterial protease that enters nerve endings and cleaves 9 C-terminal residues off a SNARE protein essential for membrane fusion called synaptosomal-associated protein of Mr = 25k (SNAP-25) (reviewed by [29]). This is highly relevant because of BoNT/A being used clinically to reduce the frequency and severity of chronic migraine attacks, though not successful in all patients [30,31]. In rats, facial injection of BoNT/A results in the cleavage of SNAP-25 in trigeminal nerves and a reduction of TRPV1 content on trigeminal fibres and cell bodies of neurons that project to the injection sites [32]. Moreover, interictal circulating CGRP levels are elevated before treatment and lowered afterwards in chronic migraine patients that respond to BoNT/A [33]. In animal pain models, the neurotoxin also inhibits the release of other neuropeptides, such as substance P and pituitary adenylate cyclase-activating peptide (PACAP), and neurotransmitters including glutamate and acetylcholine; notably, the precise sites and mechanisms of action in relation to its migraine relief remain unresolved [34]. Whilst BoNT/A in vitro blocks the CGRP release from TGNs evoked by relatively mild stimulation with a low concentration of CAP [CAP] (0.02 µM), it is progressively less effective against increasing [CAP] and a rather feeble inhibitor of CGRP release induced by 1 µM CAP [28,35]. The latter has been attributed to the recovered functionality of BoNT/A-truncated SNAP-25 in the presence of sustained large increases in [Ca^2+^]_i_ [36].

Immuno-histochemical studies have indicated that TRPV1 is found on CGRP-containing intracellular vesicles in sensory neurons [37] and co-locates with TRPA1 [26]. Evidence for the transfer of TRPA1 to the cell surface in neurons sensitised with TNFα has been accrued from its inhibition by SNAP-25 inactivating BoNT/A [26] or a recombinant chimera LC/E-BoNT/A [27], as well as knock-down of another SNARE called vesicle-associated membrane protein 1 (VAMP1) [26]. Compared with extensive knowledge of TRPV1 [23,28,38,39], the relationships between the activation of TRPA1 with different [AITC], the enhancement of their cell excitability by NGF, its potentiation of CGRP release, and susceptibility to BoNTs that inactivate VAMP or SNAP-25, are poorly defined. Therefore, these were investigated herein in cultures of TGNs isolated from neonatal rats using Ca^2+^-imaging of neurons loaded with fluo-4-acetoxymethyl ester (Fluo-4 AM) by time-lapse confocal microscopy, in combination with enzyme-linked immuno-sorbent assay (ELISA) for CGRP and Western blotting for SNAP-25 or VAMP isoforms 1/2/3. Notably, AITC stimulated Ca^2+^ entry and CGRP exocytosis in two separate tranches that were distinguished by sensitivity to [AITC], as well as dissimilar potentiation of the release by NGF. AITC could not induce increases in [Ca^2+^]_i_ as large as those evoked by 1 µM CAP; hence, in the absence of much higher [Ca^2+^]_i_ elicited by CAP, BoNT/A blocked CGRP release induced by all [AITC]. Particularly, exocytosis of the neuropeptide stimulated by either AITC or CAP was inhibited even more extensively by BoNT/DA, a new fusion protein with the neuron-acceptor binding domain of /A but that was found to cleave VAMP1/2/3. In conclusion, TGNs are excited to different extents by AITC and CAP; this confers distinct susceptibilities to BoNT/A and /DA for inhibition of stimulated exocytosis of a pain signal mediator, CGRP.

## 2. Results

### 2.1. AITC Dose-Dependently Stimulates Ca^2+^-Regulated CGRP Release from Cultured TGNs, Which Is Blocked by the TRPA1 Antagonists HC-030031 or A967079

**Figure 1:**
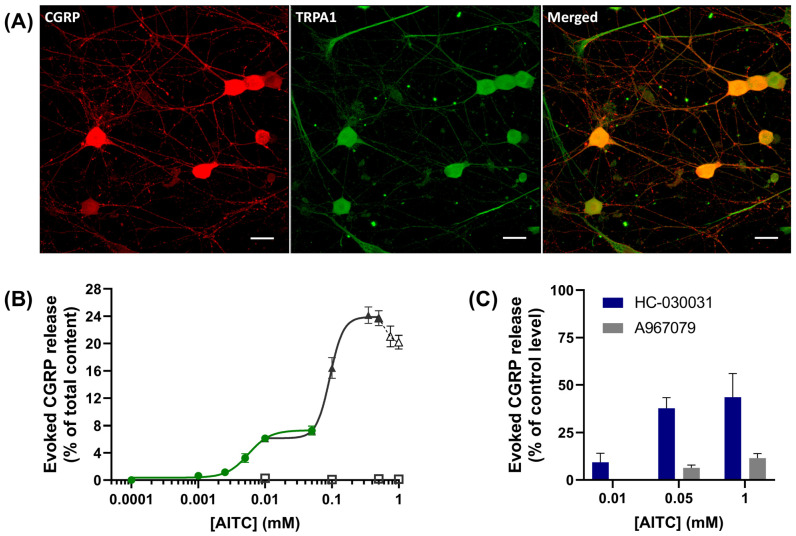
Allyl isothiocyanate (AITC) induces Ca^2+^-regulated release of calcitonin gene-related peptide (CGRP) from trigeminal ganglion neurons (TGNs) apparently via high- and low-affinity mechanisms, which are susceptible to TRPA1 antagonists HC-030031 or A967079. (**A**) Immuno-cytochemistry with confocal microscope imaging was performed on the TGNs as described in the Section 4 to detect the expression of CGRP (red) as well as TRPA1 (green) and assess their co-expression (merged). Scale bars represent 20 μm. (**B**,**C**) Cultured TGNs were exposed for 30 min to each [AITC]. Secreted and residual intracellular CGRP were quantified by ELISA as detailed in the Section 4. (**B**) The dose-response relationship between [AITC] and CGRP release (expressed as a % of the total CGRP content) was fitted with two separate four-parameter logistic functions (green, 0.0001–0.05 mM AITC and black, 0.01–0.5 mM; see Section 4). The response peaked at 0.35 mM AITC and declined for concentrations higher than 0.5 mM; data points between 0.5 and 1 mM AITC, connected with a broken line, were not included in the fitting. The quantities of CGRP released into Ca^2+^-free HEPES buffered saline (HBS) containing 2 mM EGTA by the presence of 0.01, 0.1, 0.5, or 1 mM AITC are plotted with squares; N ≥ 3, n ≥ 6. (**C**) TGNs were exposed to 100 µM HC-030031, 100 µM A967079, or vehicle only for 30 min before and during stimulation with various [AITC]. After subtraction of the spontaneous efflux, the amounts of CGRP released were calculated as detailed in the Section 4 and plotted (blue bars, HC-030031; grey bars, A967079) as a % of the requisite control (vehicle only) level elicited by each [AITC]; N = 2, n ≥ 6. Data are presented as mean ± standard error of the mean (s.e.m.); error bars are not shown where they are smaller than the associated symbol.

Immuno-cytochemistry with confocal fluorescence microscope imaging was performed to assess the co-expression of CGRP and TRPA1 in cultures of neonatal rat TGNs (Figure 1A shows representative images). As reported previously [35], immuno-labelling for CGRP (red) was detected in nearly all neuron cell bodies, albeit with variable staining intensity, and was widespread in the neuropil. Counterstaining for TRPA1 (green) revealed its presence in all the CGRP-expressing cells. To document a functional link between the activation of TRPA1 and CGRP release from sensory TGNs, the primary cultures were exposed to various concentrations of the TRPA1 agonist AITC, as described in Section 4 and Figure 1. Interestingly, a detectable increase in CGRP exocytosis (relative to the level observed in the absence of any stimulus) was elicited by as little as 0.001 mM AITC, but the amount of secreted neuropeptide plateaued between 0.01–0.05 mM, at ~7% of the total content (Figure 1B). However, raising [AITC] further provoked more neuropeptide exocytosis, reaching a maximum of ~24% of the total at 0.35 mM AITC and then declining for >0.5 mM. The data was best fit by a two-site model derived from separate curve-fitting performed on two overlapping [AITC] sub-ranges 0.001–0.05 mM and 0.01 to 0.5 mM (Figure 1B, green line, R^2^ = 0.75; black line, R^2^ = 0.86, respectively). This yielded an EC_50_ = 6 µM for an apparent higher affinity site for AITC that can elicit the release of a small fraction of CGRP and a lower affinity site EC_50_ = 93 µM responsible for an additional ~17% of the total content. Omission of extracellular Ca^2+^ prevented the CGRP release evoked by 0.01–1 mM [AITC] (Figure 1B, squares), confirming it involves Ca^2+^-regulated exocytosis. Pre-treatment of TGNs with a TRPA1 antagonist, HC-030031, inhibited ~90% of the response to 0.01 mM AITC (Figure 1C, blue bars), but the extent of antagonism was lower at higher [AITC]. An alternative TRPA1 antagonist, A967079, blocked 94 and 88% of CGRP release elicited by 0.05 and 1 mM AITC, respectively (Figure 1C, grey bars). Thus, TRPA1 is implicated in Ca^2+^-dependent CGRP release that appears to involve high- and low-affinity mechanisms for its activation by AITC.

### 2.2. AITC Provokes Bi-Phasic Ca^2+^ Signals in Cultured TGNs with Differential Stimulation of the Distinct Phases Being Dependent on the Concentration Applied

**Figure 2:**
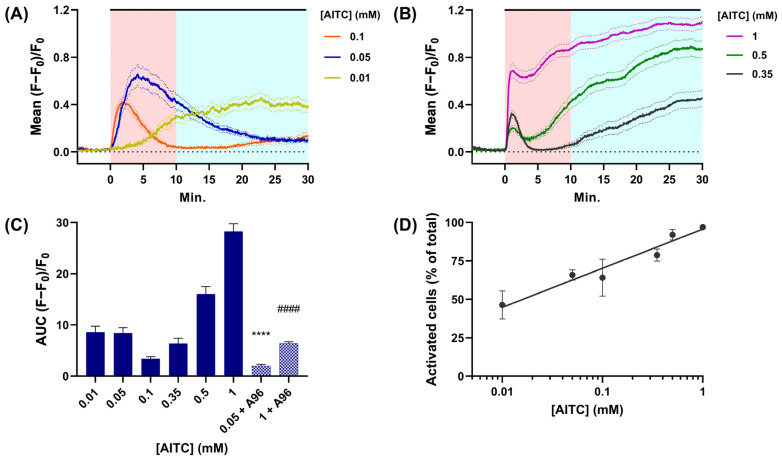
AITC elicits bi-phasic increases in [Ca^2+^]_i_ with a complex relationship to agonist concentration. Cultured TGNs were loaded with Fluo-4 AM and fluorescence intensity was recorded by time-lapse confocal microscopy. (**A**,**B**) Solid lines indicate the mean increases of fluorescence intensity (F − F_0_) evoked by various [AITC] relative to initial fluorescence (F_0_), plotted against time. Dotted lines above and below the solid traces indicate ± s.e.m. The black lines above the traces indicate the period AITC was present. (**C**) Area under the curve (AUC) of mean fluorescence change recorded over 30 min for each [AITC] in the absence (N = 3, n ≥ 100) or presence of 100 µM A967079 [(A96), N = 2, n ≥ 30]; data were analysed using unpaired *t*-test with Welch’s correction; **** *p* ≤ 0.0001, 0.05 mM AITC vs. 0.05 mM AITC + A967079; #### *p* ≤ 0.0001 1 mM AITC vs. 1 mM AITC + A967079. Error bars represent standard error. (**D**) The % of excitable cells (±s.e.m.; N = 3, n ≥ 80) that responded to different concentrations of AITC.

The neurons were loaded with a cell-penetrating Ca^2+^ sensitive dye, Fluo-4 AM, prior to monitoring responses by fluorescence microscopy before and during their exposure to AITC for 30 min (the period used previously to assess CGRP release). Independent experiments were performed for each [AITC]; only individual cells that exhibited an increase in fluorescence (F − F_0_) of more than 10 times the standard deviation (s.d.) of the baseline signal (F_0_) were used to calculate the mean increases in signal intensity (F − F_0_)/F_0_. The resultant data are plotted against time (Figure 2A,B). Whilst fluorescence remained stable at a low level prior to the addition of AITC (Figure 2A,B, white background), 0.01 mM of this TRPA1 agonist induced an increase in the mean intensity that rose steadily over the first 10 min (Figure 2A, yellow line, pink background) and plateaued over the following 10–15 min (Figure 2A, yellow line, light blue background). In neurons exposed to 0.05 mM AITC (Figure 2A, blue line), the mean signal rose more rapidly and reached a higher maximum within 5 min, but then declined over the next 25 min despite the continuous presence of the agonist. An even faster onset of the response occurred in cells exposed to 0.1 mM AITC (Figure 2A, red line), reaching a maximum value within 2 min, but this was notably lower than that observed in cells exposed to 0.05 mM AITC. This trend of faster onset (at least up to 0.35 mM) but lower maximum continued upon raising [AITC] to 0.35 and 0.5 mM (Figure 2B, grey and green line, respectively). These recordings suggest that the AITC receptor in TGNs undergoes an [agonist]-dependent desensitisation, as has been reported by others [15,16,40]. An interesting feature in neurons exposed to 0.1 or 0.35 mM AITC, a delayed slow secondary phase of fluorescence accumulation was observed after near-complete desensitisation (Figure 2A, red and Figure 2B, grey). At 0.5 mM AITC, the secondary phase started before the desensitisation of the first response had finished (Figure 2B, green line). With 1 mM AITC, the secondary signal rose so rapidly it almost completely obscured the primary response (Figure 2B, pink line). Collectively, these measurements indicate that AITC elevates [Ca^2+^]_i_ in TGNs in two phases with different concentration dependencies. A notable consequence of AITC causing activation and subsequent desensitisation is that the area under the curve (AUC) of fluorescence changes, calculated for the 0–30 min period using the GraphPad Prism^®^ software, proved similar in cells exposed to 0.01 or 0.05 mM AITC (despite their radically different profiles) but lower in TGNs exposed to 0.1 mM (Figure 2C). Only in cells treated with 0.5 and 1 mM AITC did large concentration-dependent increases in AUC become apparent. In accordance with its aforementioned blockade of CGRP exocytosis, the TRPA1 antagonist A967079 almost completely abolished the substantial increase in [Ca^2+^]_i_ induced by 0.05 mM AITC and dramatically reduced signals elicited by 1 mM AITC (Figure 2C).

### 2.3. Raising [AITC] Increases the Fraction of TGNs That Exhibit an Increase in [Ca^2+^]_i_

Another factor pertinent to the amount of CGRP release elicited is the number of cells activated by each [AITC]. To evaluate this, after exposure to AITC as detailed in Figure 2, TGNs were subsequently treated for 1 min with 1 µM CAP and then 100 mM KCl at the end of each experiment (traces not shown). Cells were counted as AITC responders if AITC caused their fluorescence intensity to increase by more than 10 × the s.d. of the baseline signal, whereas total responders incorporated those excited by any of the stimuli. Notably, the fraction of neurons responsive to AITC, calculated as a % of total responders, increased linearly for exponential increases in [AITC] from 46% for 0.01 mM to 97% at 1 mM (Figure 2D).

### 2.4. AITC Provokes Smaller Increases of [Ca^2+^]_i_ in TGNs Than CAP

**Figure 3:**
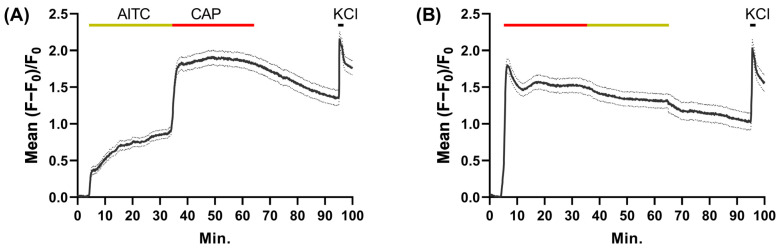
1 mM AITC induces less intense Ca^2+^ signals than 1 µM CAP in TGNs. After 5 min of baseline recordings, the cells were exposed in sequence for 30 min each to: (**A**) 1 mM AITC followed by 1 µM CAP, or (**B**) 1 µM CAP before 1 mM AITC. In both experiments after 30 min washout, 100 mM KCl was also applied for 1 min. Bars above the traces indicate the periods when the neurons were exposed to AITC (yellow), CAP (red bar), and KCl (black). N = 3, n ≥ 100. Solid and dotted black lines indicate the mean increase in fluorescence as a fraction of initial signal intensity ± s.e.m.

To compare Ca^2+^-signaling induced by 30 min stimulations with 1 mM AITC and 1 µM CAP in the same cells, TGNs were loaded with Fluo-4 AM (as described in Section 2.2) before sequential exposure to AITC followed by CAP. In addition, after exposure to CAP, the cultures were washed for 30 min and then stimulated for 1 min with 100 mM KCl to identify viable excitable cells. 1 mM AITC induced a rapid initial elevation in mean fluorescence followed by a slower phase of increase in signal intensity (Figure 3A; 5–35 min). Notably, replacing AITC with 1 µM CAP immediately led to a sharp large increment in fluorescence that was sustained throughout the period that the CAP remained present (Figure 3A; 35–65 min). This plateau decreased only slightly during the subsequent 30 min washout before the brief application of 100 mM KCl induced a third sharp rise in the mean signal. In another set of recordings (Figure 3B) with a reversal of the application sequence of AITC and CAP, the latter caused a sharper initial rise in fluorescence (Figure 3B) that was larger than the corresponding initial response to 1 mM AITC (Figure 3A). In the continued presence of CAP, after falling back slightly from the initial peak, fluorescence was sustained at a high level until this agonist was replaced with 1 mM AITC at which point the intensity declined slowly and only slightly over the next 30 min in the presence of AITC. During the subsequent washout of AITC the signal continued to decline slowly before brief exposure to 100 mM KCl induced a short sharp increase. In summary, the response provoked by 1 µM CAP was larger than that resulting from 1 mM AITC in TGNs exposed sequentially to each noxious substance one after the other, irrespective of the order of their application.

### 2.5. AITC-Induced CGRP Release Is Inhibited by BoNTs: A VAMP-Cleaving Recombinant Chimera Proved More Effective Than SNAP-25-Truncating BoNT/A

**Figure 4:**
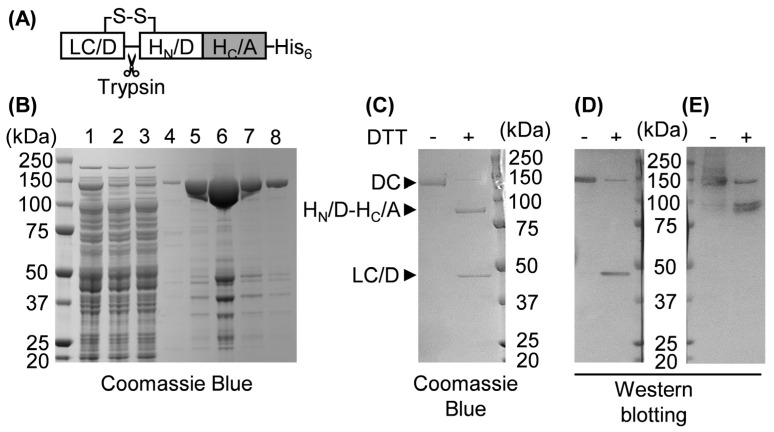
Design, purification, and characterisation of recombinant chimera DA. (**A**) Schematic of chimera DA created by recombinantly fusing LC/D and H_N_/D regions (white boxes) to H_C_/A (grey box); note that a C-terminal polyhistidine tag (His_6_) was added to allow affinity purification. S-S denotes an interchain disulphide; scissors indicate an exposed loop labile to cleavage by trypsin. (**B**) SDS-PAGE gel stained with Coomassie blue dye showing the purification of SC chimera DA from *E. coli* lysate by IMAC. Lanes: 1, cleared cell lysate; 2, flow-through from the Talon^®^ resin; 3, effluent after addition of washing buffer containing 5 mM imidazole; 4–8, fractions eluted with 500 mM imidazole. A ladder of protein standards was run in the first (non-numbered) lane and numbers to the left of each band indicate the molecular size of individual standards in kDa. (**C**–**E**) After conversion from SC to DC using Trypzean^®^ (see Section 4), the DA sample was subjected to SDS-PAGE in the absence or presence of 50 mM DTT (as indicated), followed by Coomassie Blue staining (**C**) or Western blotting (**D**,**E**) with antibodies recognising LC/D (**D**) or BoNT/A (**E**); protein ladders used in (**C**–**E**) were the same as in (**B**).

As TRPA1 and CGRP are both implicated in migraine pathology (see Section 1, [7,11]), inhibition of AITC-evoked CGRP release might offer potential as a therapeutic intervention for this debilitating condition. In this regard, prior to the assessment of AITC-evoked neuropeptide secretion, the TGNs were pre-treated with BoNTs to inactivate SNAREs that catalyse exocytotic vesicle fusion, BoNT/A to cleave SNAP-25 or a novel recombinant chimeric BoNT/DA to nullify VAMP1/2/3. The latter was created by a recombinant fusion of a gene fragment encoding the light chain (LC) plus translocation domain (N-terminal moiety of the heavy chain; H_N_) of BoNT serotype D (LC.H_N_/D) with a sequence encoding the neuronal acceptor binding domain of BoNT/A (H_C_/A) (Figure 4A). The resultant chimera DA (LC.H_N_/D-H_C_/A) was expressed as a single chain (SC) polypeptide in *E. coli* and purified via immobilised metal ion affinity chromatography (IMAC) on Talon^®^ superflow resin (Figure 4B) with a yield of ~15 mg/L of culture. Incubation of SC chimera DA with a recombinant trypsin (TrypZean^®^) transformed the majority to a disulphide-linked di-chain (DC) (Figure 4C), as reflected by the separation of the LC/D from the recombinant mosaic HC (H_N_/D-H_C_/A) only in the presence of reducing agent (50 mM dithiothreitol; DTT). The identity of the LC and the presence of BoNT/A epitopes in the HC were confirmed by Western blotting with antibodies recognising LC/D (Figure 4D) and whole BoNT/A, respectively (Figure 4E).

**Figure 5:**
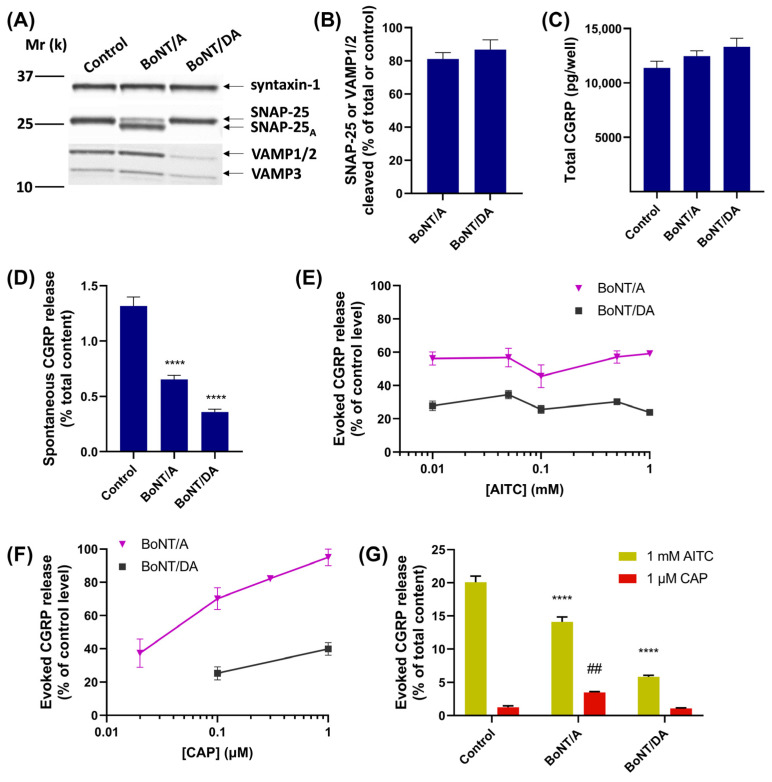
VAMP-cleaving BoNT/DA inhibits AITC- and CAP-evoked CGRP releases from TGNs more extensively than BoNT/A, which proteolyses SNAP-25. Cultured TGNs were pre-treated with 100 nM BoNT/A, BoNT/DA, or control medium for 48 h, before determining spontaneous and evoked CGRP release over sequential 30 min periods. At the end of each experiment, a cohort of cells was lysed in SDS-PAGE sample buffer and subjected to Western blotting with a mixture of three different antibodies recognising syntaxin-1 (isoforms A and B), SNAP-25 (both intact plus cleaved forms), and VAMP (isoforms 1, 2, and 3), respectively (see Section 4 for details). (**A**) Representative Western blots show that BoNT/A cleaved SNAP-25, whereas BoNT/DA proteolysed and, thereby, diminished detectable VAMPs 1/2, and 3 (note that VAMPs 1 and 2 co-migrate). Syntaxin-1 is not affected by either of the toxins, so it was used as a loading control. (**B**) The fractions of SNAP-25 or VAMP1/2 cleaved were quantified by densitometric analysis (as described in Section 4); N = 5, n = 5. Note that the weak VAMP3 signals were not analysed. (**C**) The total content of CGRP (pg/well). (**D**) Histogram displaying spontaneous CGRP release, in control cells and those treated with the BoNTs, calculated as % of the total CGRP; N ≥ 3, n = 16. (**E**) CGRP release evoked by various [AITC] for 30 min from TGNs pre-treated with BoNTs, expressed as % of requisite amounts elicited from non-intoxicated control cells; N ≥ 2, n ≥ 5. (**F**) After pre-treatment with the indicated BoNTs, TGNs were exposed to different [CAP], or (**G**) sequentially stimulated with 1 mM AITC (yellow) and then 1 µM CAP (red) for 30 min each. Evoked CGRP release is expressed as a % of control level (**F**) or in (**G**) % of the total content of this neuropeptide. Note, in (**F**) data for BoNT/A are from [41]. Data are presented as mean ± s.e.m. One- or two-way ANOVA was used in (**D**,**G**) followed by Bonferroni’s post hoc test; ## *p* < 0.01 for CAP-evoked in control vs. BoNTs; **** *p* < 0.0001 for AITC-evoked vs. BoNTs.

TGNs were initially exposed to 100 nM BoNT/A for 48 h; this cleaved 80% of the SNAP-25 (Figure 5A,B). Although no changes occurred in the total CGRP content (Figure 5C), spontaneous exocytosis of this peptide was reduced (Figure 5D). Surprisingly, BoNT/A blocked only ~40% of the CGRP release evoked by a broad range of [AITC] (Figure 5E). In view of the partial effectiveness of this SNAP-25 targeting BoNT, cells were next treated similarly with 100 nM chimera DA, resulting in VAMP1/2 being reduced by 87% compared to the level in control cells (Figure 5A,B). Accordingly, spontaneous CGRP release was reduced by 70% (Figure 5D), representing a larger reduction than observed in cells treated with BoNT/A. Moreover, BoNT/DA caused the largest inhibition of AITC-evoked CGRP release, ~75% across the whole range of agonist concentrations tested (Figure 5E). Additionally, it gave an ~80% inhibition of 0.1 µM CAP-evoked CGRP release and remained effective against 1 µM CAP (Figure 5F) that promotes much larger increases in [Ca^2+^]_i_ [28]. This contrasts with the inhibition afforded by BoNT/A being restricted to lower CAP concentrations (Figure 5F). These results show that the VAMP-cleaving BoNT gave a more complete inhibition of CGRP release elicited by AITC or CAP (see Section 3).

### 2.6. Depletion of CGRP from TGNs by AITC Stimulation Prevents CAP from Evoking Further Release

To ascertain whether AITC and CAP stimulate CGRP exocytosis from the same TGNs, exposure to AITC was followed by CAP, and the amounts of CGRP released during stimulation with each of the TRP channel agonists were quantified. During 30 min exposure to 1 mM AITC, 20% of the total CGRP present was exocytosed (Figure 5G, control), leaving 80% retained inside the cells. Nevertheless, subsequent exposure to 1 µM CAP proved unable to stimulate more than a minimal amount of additional CGRP release, meaning either that pre-exposure to 1 mM AITC depleted virtually all the CGRP that can be mobilised by 1 µM CAP or this prior treatment of CAP-excitable TGNs with 1 mM AITC causes extensive desensitisation of TRPV1 (a less likely scenario, see Figure 3 and Section 3). Either way, it can be deduced that the vast majority of TGNs co-expressing CGRP and TRPV1 very likely express TRPA1 too.

### 2.7. In BoNT/A-Treated Neurons CAP Elicits a Fraction of CGRP Exocytosis When Applied after AITC

CGRP release upon sequential stimulation by AITC and then CAP was next investigated in TGNs pre-intoxicated with BoNT/A. As noted in Section 2.5, such pre-treatment only partially inhibited the amount of CGRP released during 30 min exposure to AITC; BoNT/A-treated cells released 14% of their CGRP, a reduction of 6% compared to non-intoxicated control cells (Figure 5G). Upon subsequent stimulation with 1 µM CAP some additional CGRP was released; though this only represented 3.5% of the total content, it seems that CAP could mobilise a small fraction of the releasable neuropeptide that was unresponsive to AITC after BoNT/A intoxication. This accords with the inability of BoNT/A to block 1 µM CAP-evoked CGRP release from TGNs (Figure 5F). Moreover, as AITC and CAP stimulate the same population of cells (see Section 2.3 and Section 2.6), the recovered exocytosis cannot be due to the recruitment by CAP of cells that are unresponsive to AITC. By contrast, BoNT/DA inhibited responses to 1 mM AITC more extensively, with CAP being unable to elicit any significant amount of CGRP release from TGNs intoxicated with this chimera (Figure 5G).

### 2.8. NGF Enhances the Release of CGRP Evoked by Low [AITC]

**Figure 6:**
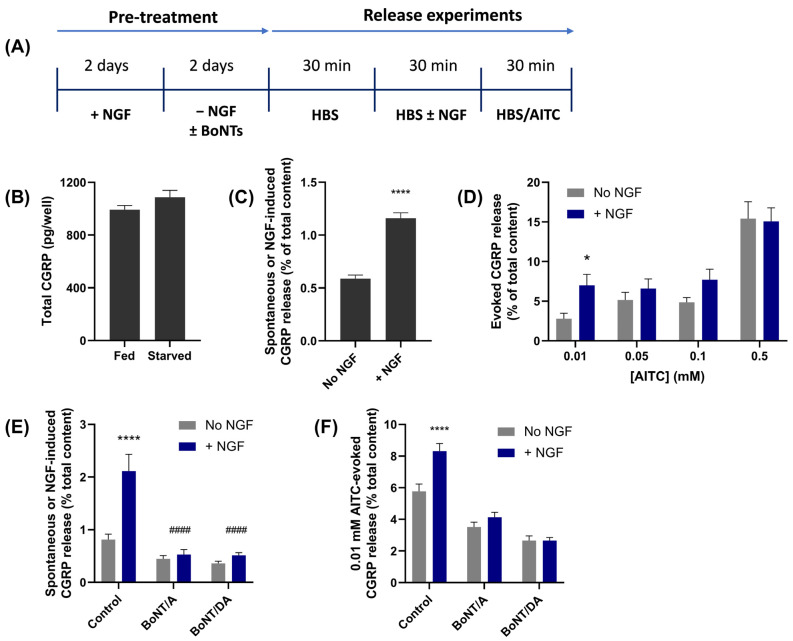
BoNT/A or /DA inhibits both the induction by NGF of CGRP exocytosis and its enhancement of 0.01 mM AITC-evoked neuropeptide secretion. (**A**) Timeline for manipulations applied to cultured TGNs: NGF feeding and withdrawal, exposure to BoNTs, and steps performed to determine the effect of acute NGF (100 ng/mL) on CGRP release. (**B**) Similar amounts of CGRP were detected in neurons grown for 4 days in the presence of NGF (Fed) or 2 days with NGF followed by two days without (Starved). (**C**–**F**) Histograms showing the levels of CGRP release (as a % of total cell content) during 30 min exposure of 2-day NGF-starved TGNs: (**C**) to HBS in the absence (No NGF) or presence of 100 ng/mL NGF (+NGF); (**D**) to various [AITC] from TGNs that had previously been exposed to HBS without (grey bars) or including 100 ng/mL NGF (blue bars) just prior to stimulation with the noxious substance. Two-tailed Welch’s tests were applied to compare responses between NGF-free and −30 min treated groups at each AITC concentration, * *p* < 0.05; (**E**) to HBS only (grey bars) or HBS + 100 ng/mL NGF (blue bars) from TGNs that had been deprived of NGF for 48 h in the absence (Control) or presence of 100 nM of the indicated toxins; two-way ANOVA followed by Bonferroni’s post hoc test was used; **** *p* < 0.0001 for pre-treatment with NGF vs. no NGF; #### *p* < 0.0001 for NGF-induced in control cells vs. those pre-treated with the indicated BoNTs; (**F**) to 0.01 mM AITC from control or BoNT-pre-treated TGNs that had just been exposed for 30 min to 100 ng/mL NGF (blue bars) or HBS only (grey bars). The significance was tested by two-way ANOVA followed by Bonferroni’s post hoc test; **** *p* < 0.0001 AITC-evoked CGRP release after pre-treatment with NGF vs. no NGF. N ≥ 2, n = 6 for /A, n = 10 for /DA.

NGF is one of several factors found to accumulate in cerebrospinal fluid in patients with chronic migraine [21] that have been shown to potentiate the activity of some TRP channels [42], so its effect on AITC-evoked CGRP release in TGNs was examined. There is a requirement for NGF in the culture medium for the survival in vitro of TGNs isolated from neonatal rats, but its continuous presence would mask any effect on the secretion of CGRP. Therefore, a protocol for the removal and re-addition of NGF was adopted [28], as illustrated in Figure 6A. Newly-isolated TGNs were grown for 2 days in the presence of 50 ng/mL NGF to establish an attachment to the substratum and facilitate the development of an immature neuropil. This was followed by transfer into a fresh medium lacking NGF and supplemented with anti-NGF antibodies to neutralise any residual traces of the neurotrophin. These TGNs were cultured for another 2 days in this NGF-free medium before assessing the effect of acute re-exposure to NGF (100 ng/mL) on CGRP release, and on the additional exocytosis evoked by various [AITC]. As found previously [28], growth without the neurotrophin for 2 days did not reduce the amount of CGRP expressed in these cells (Figure 6B). Exposure to NGF for 30 min induced a small but significant (*p* < 0.0001) increase in CGRP secretion over spontaneous release compared to the amount recorded in the absence of NGF (Figure 6C). Interestingly, 30 min incubation of TGNs with the neurotrophin prior to stimulation with AITC significantly (*p* = 0.03) enhanced CGRP secretion but only at the lowest agonist concentration, 0.01 mM (Figure 6D). Although the amounts of CGRP release evoked by 0.05 or 0.1 mM AITC were also augmented slightly by the brief treatment with NGF, these changes were not significant. NGF showed no effect on the amount of CGRP exocytosis stimulated by 0.5 mM AITC (Figure 6D).

### 2.9. CGRP Release Induced by NGF and Its Enhancement of Secretion Evoked by Low [AITC] Are Both Blocked by BoNT/DA or /A

To evaluate if VAMP1/2/3 and SNAP-25 are involved in the influence of NGF on CGRP release, the cultured TGNs were pre-treated with 100 nM of BoNT/DA or /A during the 2-day NGF withdrawal period (Figure 6A). The release assay was then performed as described above. /DA prevented the induction by NGF of CGRP exocytosis (Figure 6E), confirming that VAMP1/2 and/or 3 is involved in this process; furthermore, the inhibition obtained with /A reaffirms [28] a requirement for SNAP-25. Notably, BoNT/DA and BoNT/A both reduced CGRP release evoked by 0.01 mM AITC from NGF-starved cells that were not acutely exposed to the growth factor (Figure 6F, grey bars). More strikingly, each toxin prevented augmentation of 0.01 mM AITC-evoked CGRP release by the brief re-introduction of NGF relative to that observed in control cells. Thus, VAMP1/2/3 and SNAP-25 are essential mediators of TGN sensitisation by NGF to AITC, implicating SNARE-mediated membrane fusion in the process.

## 3. Discussion

Regarding molecular mechanisms that contribute to pathological pain, there has been great interest in TRP channels that play key roles in the excitation of nociceptors, and neuropeptides such as CGRP that are released consequently [3,5,7,11,43]. Nevertheless, the details of how these processes are linked to each other and pain signaling are still unclear. Here, AITC was used to activate TRPA1 in cultures of rat TGNs, and the resultant increases in [Ca^2+^]_i_ were correlated for the first time with the extent of CGRP release. Notably, this revealed that there are two mechanisms for AITC to evoke CGRP exocytosis implicating distinct affinities of this electrophilic agonist for the TRPA1 channel (Figure 1B). Furthermore, the effect of NGF, a neurotrophin implicated in inflammatory pain, on AITC-induced CGRP release was found to enhance significantly only CGRP release elicited by a low [AITC], 0.01 mM, indicating a selective action via the more sensitive mechanism. Finally, with a view to the expected therapeutic benefits of lowering CGRP release and suppressing its enhancement by NGF [3,44,45], inhibition of both processes by BoNT/A was examined, as well as by a recombinant variant BoNT/DA that retains the neurotropism of BoNT/A but targets VAMP1/2 and 3 rather than SNAP-25. Notably, this unveiled that whilst both BoNTs attenuated sensitisation of TGNs by NGF, the VAMP-cleaving /DA gave a more extensive reduction of AITC-evoked CGRP release.

The complex concentration-dependency of AITC-evoked CGRP release accords with the intricacy of TRPA1 activation by electrophiles. The initial stimulation at low concentrations of AITC that elicits the release of ~7% of the total CGRP content (Figure 1B) is compatible with the AITC sensitivity of cysteines (C621, C641, and C655) that occupy a pocket in the so-called ‘coupling domain’ on the cytoplasmic side of TRPA1 [12,13,46]. A triple mutant (TRPA1-3C [12]; C621S, C641S, and C655S) exhibited a greatly reduced activation by AITC < 0.1 mM. Covalent modification by small electrophiles such as AITC of C621 and C655 stabilises an open pocket conformation that, in turn, facilitates the opening of the TRPA1 channel pore. The second and larger increment of CGRP release would seem to imply a reaction with TRPA1 at another site. In this regard, TRPA1-3C has been shown to be activated upon prolonged exposure to high [AITC] (>0.1 mM); this seems to involve modification of K708 because mutation of the latter rendered the channel (TRPA1-3C-K708R)-insensitive to the electrophile [12]. Although exposure to such high [AITC] for a prolonged time (30 min) will modify other cell proteins [13], including TRP channels that may consequently be opened [47], even the CGRP release induced by 1 mM AITC was inhibited by either of two selective TRPA1 antagonists, HC-030031 or A967079 (Figure 1C). Thus, the big increment in CGRP exocytosis evoked by the higher [AITC] may be attributable to such further activation of TRPA1, but a small contribution from other channels cannot be excluded. Confirmation of this model will require analysis of CGRP release from sensory neurons in which either the TRPA1-3C or TRPA1-3C-K708R mutant has replaced the wild-type TRPA1, a challenging endeavour outside the scope of this study.

Notably, TRPA1-3C stimulated by high [AITC] displays the same unitary conductance as the wild-type channel at low [AITC] [12]. If the heterologous expression of TRPA1 in HEK cells faithfully reflects the activity of this channel in neurons, it seems unlikely that alteration of Ca^2+^ conductance could underlie the large increase in CGRP release at high [AITC]. So, the relationship between [AITC] and [Ca^2+^]_i_ during prolonged exposure to the agonist was investigated in TGNs loaded with Fluo-4 AM. When exposed to the lowest [AITC], 0.01 mM, there was a slow progressive rise in fluorescence (Figure 2A). As this is reminiscent of TRPA1 activation by low concentrations of an electrophile, it appears attributable to the involvement of a chemical reaction that would slowly yield modified cysteines and, thereby, open channels [12]. Collision theory predicts that increasing the concentration of one of the reactants (AITC) accelerates the chemical reaction and, thereby, the build-up of the product (TRPA1 with modified cysteines and an open channel). Accordingly, exposing dye-loaded TGNs to 0.05 mM AITC induced a much faster increase in fluorescence than observed with 0.01 mM (Figure 2A). However, after a few minutes of exposure to 0.05 mM AITC, the intensity of fluorescence started to decline despite the agonist’s continued presence; this invokes another well-established characteristic of TRPA1, Ca^2+^-dependent desensitisation [14,15,16]. As Ca^2+^ permeates through the channel, it is captured by a cytoplasmic Ca^2+^-binding site proximal to the pore [15,16]; this initially potentiates TRPA1 channel conductance, but is followed by a delayed reduction in conductivity, termed desensitisation [15]. Interestingly, a single mutation E788S in the Ca^2+^-binding site is sufficient to prevent Ca^2+^-mediated potentiation of TRPA1 activity, whilst two additional mutations Q791S and N805S are required to prevent Ca^2+^-mediated desensitisation [16]. This delayed TRPA1 desensitisation could explain the contrary effects of increasing [AITC] on fluorescence intensity (Figure 2A); an initial rise in fluorescence is accelerated with rising [AITC], but signals crest and then decline sooner as desensitisation ensues. A consequence of such desensitisation is that the integrated Ca^2+^-signal (AUC of time course) does not increase upon raising [AITC] from 0.01 to 0.05 mM (Figure 2C); accordingly, similar amounts of CGRP exocytosis were evoked (Figure 1B). When AITC was increased to ≥0.1 mM, secondary delayed elevations of fluorescence appeared (Figure 2B,C). These might correspond to slower TRPA1 activation by AITC reacting with the weaker amine nucleophile, K708 [16]. So, it is tempting to speculate that the secondary response signal is minimal at low [AITC], delayed at intermediate [AITC], and accelerated by high [AITC], the pattern observed experimentally. Notably, channels activated by K708 modification are resistant to Ca^2+^-dependent desensitisation [16]. Hence, the large and sustained increases in the time-integrated Ca^2+^-signals observed for TGNs exposed to 0.35, 0.5, and 1 mM AITC (Figure 2B,C), and a larger fraction of the cells that responded (Figure 2D). This accounts for the large amounts of CGRP exocytosis evoked by 0.35 and 0.5 mM AITC, though the extent of exocytosis in response to 1 mM AITC was somewhat lower. A similar outcome has been observed for CGRP release elicited from TGNs by high [CAP] [28]. It seems that very large increases in [Ca^2+^]_i_ are sub-optimal for CGRP release, which might also explain why 0.05 mM AITC evoked less CGRP exocytosis than 0.1 mM despite a more robust Ca^2+^ signal; this could also account for neuropeptide release peaking at 0.35 mM AITC even though 0.5 and 1 mM induced larger Ca^2+^ signals.

**Figure 7:**
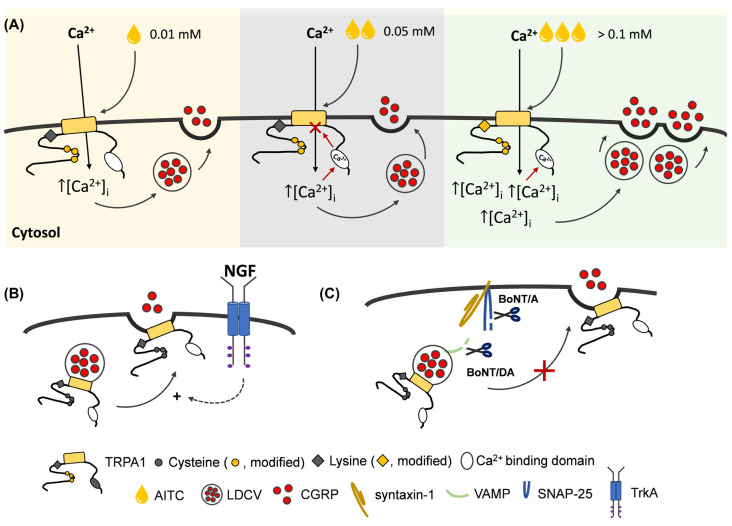
The hypothetical model for [AITC]-dependence of CGRP release from neonatal rat TGNs in vitro, the enhancement of low [AITC]-evoked exocytosis by acute NGF and the blockade of these pain-related processes by BoNTs that inhibit membrane trafficking. (**A**) At low concentrations of AITC (0.01 mM; yellow background), in accordance with those reported to activate TRPA1 by the modification of strongly nucleophilic cysteine residues (●) in the channel [12], a relatively small increase in [Ca^2+^]_i_ evokes a modest amount of CGRP exocytosis. Moderate increases to 0.05 mM AITC (grey background) cause [Ca^2+^]_i_ to rise more rapidly to higher levels initially, but do not elevate the AUC of Ca^2+^ signal over the full 30 min time course; this may be reconciled with the known delayed desensitisation of TRPA1 mediated by an intracellular Ca^2+^-binding domain proximal to the channel pore [15,16]. High [AITC] (>0.1 mM; green background) induces large increases in [Ca^2+^]_i_, which seems consistent with the modification of a weaker nucleophilic K708 (◆) that causes a non-desensitising TRPA1 activation [16], thereby, provoking the exocytosis of a larger amount of CGRP. At >0.35 mM AITC, large increases of [Ca^2+^]_i_ are elicited without corresponding increments in CGRP exocytosis, likely due to the cation concentration exceeding the optimum level for catalysis of membrane fusion. (**B**) NGF induces a small amount of CGRP release and it is presumed that TRPA1 on the peptidergic granules [26] is trafficked to the plasma membrane. This enhances subsequent responses to 0.01 mM AITC, but not higher concentrations due to various possible reasons; for example, increased Ca^2+^ entry might be attenuated by faster Ca^2+^-dependent desensitisation, small NGF-induced enhancements might be obscured by large AITC-evoked signals at high agonist concentrations or increases in plasma membrane TRPA1 might be non-productive at high [AITC] because the optimum [Ca^2+^]_i_ for triggering CGRP release has been exceeded. (**C**) CGRP exocytosis and the trafficking of TRPA1 to the plasma membrane are inhibited by BoNT/A and BoNT/DA that cleave (scissors) SNAP-25 and VAMP1/2/3, respectively. Some clinical evidence supports the notion that BoNT/A can provide migraine relief to patients by reducing excess CGRP exocytosis [33]. No VAMP-cleaving toxin is licensed for migraine treatment but a few clinical trials have been performed, with some evidence of symptom improvements [48]. Nevertheless, a definitive mechanism of action for BoNT treatment of migraine remains elusive.

In summary, complex patterns of increases in [Ca^2+^]_i_ were observed in TGNs exposed to different [AITC], but these can be nominally reconciled with current knowledge of the multi-site chemical activation and desensitisation of TRPA1 (Figure 7A). Consequently, Ca^2+^ influx is limited in amount and duration at low [AITC], despite initial activation of the signal, due to desensitisation restricting the stimulation of CGRP release to a relatively low level (~7%). By contrast, relatively high [AITC] known to additionally stimulate TRPA1 by a secondary mechanism resistant to desensitisation [12] elicits large and persistent elevations in [Ca^2+^]_i_ that evoke a substantial extra increment in CGRP exocytosis, but very large increases in [Ca^2+^]_i_ are sub-optimal.

The big, sustained rise in [Ca^2+^]_i_ induced by CAP after AITC pre-treatment (Figure 3A), is not compatible with the proposal that cross-desensitisation of TRPV1 by AITC impairs CAP-evoked CGRP release [49]. An alternative possible explanation is that pre-exposure to 1 mM AITC depletes the cells of readily releasable CGRP (Figure 5G, control). To investigate this notion advantage was taken of the poor inhibition by BoNT/A of CGRP release evoked by 1 µM CAP [28,35]. Pre-intoxication with BoNT/A reduced the amount of CGRP released in response to 1 mM AITC, relative to untreated control cells (Figure 5G), so the releasable reserve was depleted but only partly. Subsequent stimulation of the BoNT/A-intoxicated TGNs with 1 µM CAP was able to stimulate the exocytosis of a residual portion of releasable CGRP (Figure 5G). This ability of CAP to release the remaining CGRP corroborates the Ca^2+^ imaging data to suggest that pre-exposure to AITC does not cause extensive cross-desensitisation of TRPV1 in TGNs but, rather, depletes the neurons of releasable CGRP.

Neurotrophins such as NGF might potentiate TRPA1 activity by the activation of phospholipase C and degradation of phosphatidyl inositol polyphosphates (PIPs) that directly bind to TRPA1 channels and suppress their activity [13,16]. Alternatively, NGF may mobilise to the cell surface intracellular reserves of TRPA1 residing on CGRP-containing secretory granules (Figure 7B), similar to that of observed with TNFα [26]. Here, using a protocol that can reliably detect an enhancement of TRPV1 activity by NGF [28], it was found that NGF pre-treatment furnishes a modest augmentation of AITC-evoked CGRP release but only for low [AITC] (Figure 6D). The lack of elevation of CGRP release evoked by 0.5 mM AITC is probably due to the maximum stimulation of CGRP release having been reached even in the absence of NGF; a similar outcome was observed previously for the enhancement by NGF of CGRP release evoked by low but not high [CAP] [28]. The inferred TRPA1 trafficking could also underlie AITC self-potentiation of cell excitability to repeated application, and this has been attenuated by tetanus toxin [50] which cleaves VAMP1/2/3. The likely involvement of the channels’ delivery to the cell surface from intracellular reserves was highlighted by pre-intoxication of the TGNs with BoNT/A or BoNT/DA because both inhibitors of SNARE-mediated membrane fusion prevented the NGF enhancement of 0.01 mM AITC-evoked CGRP release (Figure 6F) as well as its induction of exocytosis (Figure 6E).

BoNTs potentially offer a prospect of analgesia through specific inhibition of membrane fusion, encompassing but not restricted to TRP channel trafficking and CGRP release (Figure 7C). The exocytosis of additional neuropeptides and neurotransmitters may also be attenuated by BoNT/A and the trafficking of other pain-signalling proteins could also contribute to migraine [34]. BoNT/A has been approved and is used clinically with some success for intransigent chronic migraines and off-label for other severe headaches [48], being recommended as a third-level preventative treatment if first-line (beta-blockers, GABA_A_-agonists or angiotensin II receptor antagonists) and second-line (amitryptiline [a tricyclic anti-depressant], flunarizine [Ca^2+^ antagonist] or sodium valproate [an anti-convulsant]) options prove unsatisfactory [51]. Interestingly, preliminary clinical evidence suggests that therapies combining BoNT/A injections with antibodies that suppress CGRP signaling are more effective than either treatment alone (reviewed by [52]). A mechanistic basis is lacking, but these observations seem to indicate that neither BoNT/A nor antibody therapy alone can fully antagonise CGRP signaling in vivo. It was found here that BoNT/A caused only a partial inhibition of AITC-evoked CGRP to release in vitro (Figure 5E) despite extensive proteolysis of SNAP-25 (Figure 5A,B) to an extent previously found to result in lowering CGRP release evoked by K^+^ depolarisation, bradykinin or low [CAP] [28,35]. Inhibition of CAP-evoked CGRP release by BoNT/A is overcome by raising [CAP], which has been attributed to this causing larger and more persistent increases in [Ca^2+^]_i_ [28,36]. However, persistent high [Ca^2+^] is unable to account for the partial inhibition of AITC-evoked CGRP release by BoNT/A, as a similar incomplete blockade was observed over a broad series of [AITC] (Figure 5E) that elicited a diverse range of [Ca^2+^]_i_ signals (Figure 2A,B). Moreover, even 1 mM AITC proved incapable of raising [Ca^2+^]_i_ to anywhere near the level induced by 1 µM CAP (Figure 3). By contrast, in TGNs pre-treated with BoNT/DA, evoked CGRP release was consistently inhibited by ~70% at all [AITC] tested (Figure 5E), in closer alignment with the observed 85% reduction of intact VAMP1/2 (Figure 5A,B). This confirms that AITC-evoked CGRP exocytosis is SNARE-mediated, so the reason for relatively weak inhibition by BoNT/A remains unknown. It might be that the residual intact SNAP-25 (~20% of the total content) is disproportionately capable of mediating AITC-evoked CGRP release.

## 4. Materials and Methods

### 4.1. Materials

NGF 2.5S (N-100), anti-NGF (AN-240) and anti-TRPA1 (ACC-037) antibodies, TRPA1 antagonists A967079 (A-225) and HC-030031 (H-105) were supplied by Alomone Labs (Jerusalem, Israel). Anti-CGRP antibodies (ab81887) were purchased from Abcam (Cambridge, Cambs., UK). Culture 48-wells plates and ProLong^TM^ Glass Antifade Mountant were obtained from Thermo Fisher Scientific (Winsford, Cheshire, UK). Collagenase, Dispase^®^, B-27^TM^ Supplement, lithium dodecyl sulphate (LDS) sample buffer, and 12% BOLT™ Bis-Tris polyacrylamide gels were bought from Bio-Sciences (Dublin, Ireland). Monoclonal antibodies specific for SNAP-25 (clone SMI-81; SMI-81R) or syntaxin-1 (clone HPC-1; S0664) were supplied by Covance (now Labcorp Drug Development, Princeton, NJ, USA) and Merck (Arklow, Ireland), respectively, whilst polyclonal anti-VAMP1/2/3 (104 102) was from Synaptic Systems (Göttingen, Germany). An antibody that reacts with the LC of BoNT/D was a kind gift from Dr. Leonard Smith (USAMRIID), and an anti-serum to BoNT/A [53] was provided by Zymed Laboratories Inc. (San Francisco, CA, USA). Goat secondary antibodies reactive with a mouse (A3688) or rabbit (A9919) IgGs and conjugated with alkaline phosphatase (AP), or horseradish peroxidase were supplied by Merck. Alexa Fluor 488 (A21206) or 633 (A21202) conjugated secondary antibodies were from Invitrogen (Fisher Scientific, Loughborough, Leics., UK), Western blotting reagents, polyvinylidene fluoride membrane (PVDF) and Bio-Rad protein standards were bought from AccuScience (Kildare, Ireland). Enzyme-linked immuno-sorbent assay (ELISA) kits for the detection and quantification of CGRP were procured from Bertin Technologies (Montignyle Le Bretonneux, France). BoNT/A was purchased from Metabiologics (Madison, WI, USA). PD-10 columns and all other reagents used were obtained from Merck.

### 4.2. Gene Fusion, Expression, and Purification of SC Chimera DA Followed by Its Conversion to a DC by Restricted Proteolysis

The engineering of recombinant BoNTs and protein expression in genetically modified *E. coli* was approved by the Environmental Protection Agency of Ireland (project no. G0167) and notified to the Irish Health and Safety Authority. This work was performed in a containment level 3 laboratory. To create chimera DA, a synthetic gene with codons optimised for expression in *E. coli* of LC.H_N_/D domains was fused to the synthetic gene encoding H_C_/A. The resultant gene fusion encoding the protein chimera DA (LC.H_N_/D-H_C_/A) was subsequently cloned into a previously-reported construct, replacing a chimera BA gene [54]. This yielded a plasmid encoding chimera DA with a C-terminally fused His_6_ tag for affinity chromatography purification. The latter was transformed into *E. coli* BL21.DE3 strain and its encoded proteins are expressed using an auto-induction medium [55], following a previously-established protocol for BoNT/EA [53]. Briefly, bacteria grown overnight in Luria-Bertani broth were inoculated (1:1000 *v*/*v*) into ZYP-5052 medium [55] and cultured at 37 °C for ~4 h at 220 rpm/min before reducing the temperature to 22 °C for 20 h for auto-production of chimera DA. The harvested cells were resuspended in lysis buffer (20 mM HEPES, 145 mM NaCl, pH 8.0) supplemented with a protease inhibitor cocktail III (1:200 *v*/*v*) and 1000 units of Benzonase^®^ nuclease. Cells were lysed at 4 °C for 1 h by adding lysozyme to a final concentration of 1 mg/mL. After two freeze-thaw cycles, the cell lysate was clarified by centrifugation at 18,000× *g* for 1 h. The resultant supernatant was subjected to IMAC on Talon^®^ superflow resin following the manufacturer’s protocol. The eluted chimera DA was buffer-exchanged into 20 mM HEPES, 145 mM NaCl, pH 7.4 using a PD-10 desalting column and nicked by TrypZean^®^ (2 µg per mg of chimera DA) for 1 h at 22 °C before the addition of soybean trypsin inhibitor and storage of the sample at −80 °C. The purified chimera DA was monitored by SDS-PAGE on precast NuPAGE 4–12% Bis-Tris gels, followed by protein staining with Coomassie Blue or Western blotting with rabbit polyclonal antibodies recognising LC/D (1:1000) or BoNT/A (1:3000). After washing and incubation with horseradish peroxidase labelled donkey anti-rabbit IgG (1:5000), blots were developed with 3,3′,5,5′-tetramethylbenzidine substrate. Samples were visualized and digitally photographed using the G:BOX gel imaging system (Syngene, Cambridge, UK).

### 4.3. Isolation and Culturing of Rat TGNs

TGNs were dissected from 3 to 6 day-old Sprague Dawley rat neonates, digested to break down connective tissues, and spun through the Percoll^®^ gradient as described [28]. The cells were re-suspended in Dulbecco’s Modified Eagle Medium (DMEM) containing 10% (*v*/*v*) foetal bovine serum, 1% (*v*/*v*) penicillin-streptomycin, 2% (*v*/*v*) B-27^TM^ Supplement, and 50 ng/mL 2.5 S NGF. TGNs were seeded at a density of ~20,000–30,000 neurons per well in 48-well plates (for release experiments) or 30,000–35,000 on coverslips (Ca^2+^ imaging) that had been pre-coated with poly-L-lysine (0.1 mg/mL) and laminin (20 µg/mL). To suppress the growth of dividing (i.e., non-neuronal) cells, 10 μM cytosine arabinoside (Ara-C) was added on day 1 and included for 4 consecutive days; the medium was changed every day unless otherwise specified.

### 4.4. Immuno-Cytochemistry

TGNs grown on 13 mm glass coverslips for 4 days in the presence of 50 ng/mL NGF were prepared for immune-cytochemistry as previously described [35]. Briefly, the neurons were washed once with Dulbecco’s phosphate buffer saline (DPBS) and fixed for 30 min with 3.7% paraformaldehyde in the same buffer before permeabilisation with 0.5% (*v*/*v*) Triton X-100 in DPBS, supplemented with 0.1% (*w*/*v*) BSA. After blocking with 10% (*v*/*v*) donkey serum in DPBS for another 30 min, the cells were exposed for 2 h at room temperature to mouse antibodies for CGRP (1:500 dilution) and rabbit anti-TRPA1 (1:200) in DPBS containing 10% (*v*/*v*) donkey serum. After washing with 0.1% (*v*/*v*) Triton X-100 in DPBS, bound primary antibodies were detected using a mixture of donkey anti-mouse IgG and donkey anti-rabbit IgG conjugated with Alexa fluor^®^ 633 and 488 dyes, respectively, applied for 1 h at room temperature. After another round of washing, the stained coverslips were mounted with ProLong^TM^ Glass Antifade Mountant and imaged on a Zeiss Observer Z1-LSM710 confocal microscope. Images were acquired through an EC Plan-NEOFLUAR oil objective with 40× magnification and 1.3 numerical aperture, using Zen Black 2.3 software (Carl Zeiss, Oberkochen, Germany).

### 4.5. NGF Withdrawal from TGNs and Treatment with BoNTs

After 2 days in vitro, cells were washed thrice with 0.5 mL per well of DMEM-based standard medium but lacking NGF and containing 500 ng/mL anti-NGF antibodies, as well as 10 μM Ara-C. For the next 2 days, TGNs were maintained in this starvation medium or with the inclusion of 100 nM BoNT/A or BoNT/DA; then, spontaneous, NGF-induced, and AITC-stimulated CGRP release were quantified under the different conditions specified.

### 4.6. Incubation of TGNs to Monitor CGRP Release and Its Quantification by ELISA

Release of CGRP was determined as previously described [28] using 0.1 mL aliquots added to 96-well plates; ELISA was performed following instructions for the kit. The spontaneous release values were subtracted from those obtained with AITC or CAP to yield the evoked component. Briefly, TGNs were incubated for 30 min at 37 °C with 0.25 mL per well of HEPES buffered saline [HBS, mM: 22.5 HEPES, 135 NaCl, 3.5 KCl, 1 MgCl_2_, 2.5 CaCl_2_, 3.3 glucose, and 0.1% bovine serum albumin (BSA), pH 7.4] and except for the measurement of spontaneous release, stimulants were added to HBS. Note that for some experiments, CaCl_2_ was omitted from the HBS and replaced with 2 mM EGTA, as detailed in the relevant Figure legends. For stimulation with AITC or CAP and antagonising TRPA1 with A967079 or HC-030031, working dilutions in HBS were prepared on the day of use from a 1 M stock of AITC in DMSO, 100 mM CAP in ethanol, 100 mM A967079 or 100 mM HC-030031 in DMSO, which were stored at −20 °C. As a control for the solvent, 0.1% DMSO or ethanol was included in the HBS as a vehicle during the first 30 min incubation to allow the spontaneous release of CGRP to be monitored. At the end of each experiment, all remaining fluid was aspirated, and the cells dissolved in 1% (*v*/*v*) Triton X-100/HBS. All aliquots were removed after the various incubations, and the detergent-lysed cells were centrifuged (20,000× *g*) for 1 min at 4 °C to remove insoluble matter, and the supernatants were stored at −20 °C until the day of assay.

### 4.7. Western Blotting and Quantification of SNAP-25 and VAMP1/2/3 Cleavage

Cells were washed with HBS, dissolved in an LDS sample buffer, and incubated at 95 °C for 5 min prior to electrophoresis on 12% BOLT™ polyacrylamide SDS gels. Western blots were performed with three antibodies selective for syntaxin-1 (raised in mouse, 1:2000); SNAP-25 (mouse monoclonal which recognises the intact and truncated protein, 1:3000), or VAMP1/2/3 (rabbit polyclonal, 1:1000) for either 1 h. at room temperature or overnight at 4 °C. Following additional wash steps, the membranes were exposed to AP-conjugated secondary antibody (1:10,000) for 1 h. at room temperature. Bound immunoglobulins were visualised by the development of a coloured product during incubation with 5-bromo-4-chloro-3-indolyl phosphate (0.17 mg/mL), and nitroblue tetrazolium chloride (0.33 mg/mL) in substrate buffer (mM: 100 Tris, 100 NaCl, 5 MgCl_2_, pH 8.5). Images of the membrane were digitised using a G:BOX; their densitometric analysis was performed using ImageJ software (ImageJ 1.53e, National Institutes of Health, USA), with the resultant data normalised to the signals for total SNAP-25 (cleaved and intact) or VAMP1/2/3 in non-toxin treated control samples, with adjustments for protein loading by standardisation to syntaxin.

### 4.8. Intracellular Ca^2+^ Imaging

TGNs were prepared and cultured as described in 4.3 but were plated on 13 mm glass coverslips coated with poly-L-lysine and laminin. After 4 days in culture, cells were washed with HBS supplemented with a low (10 µg/mL) of BSA (HBS-LB) and loaded with 3 µM Fluo-4 AM in the presence of 0.02% pluronic F-127 acid for 20–30 min at 37 °C. Coverslips were then placed in a superfusion chamber (RC-25; Warner Instruments, Holliston, MA, USA) mounted on the stage of a Zeiss LSM710 confocal microscope and left to equilibrate for 10 min with 2 mL/min continuous perfusion of HBS-LB. Confocal imaging was performed at ambient temperature (22–26 °C) using a 488 nm argon laser and 20× magnification objective (EC Plan-NEOFLUAR/0.5 NA) at a 0.33 Hz frame rate. Baseline fluorescence was recorded for 5 min before switching to HBS-LB containing AITC or CAP, which was added from appropriately-diluted stock solutions prepared in a vehicle on the day of use, and recordings continued in their presence for 30 min At the end of each experiment, after washing out the agonists, TGNs were stimulated with 100 mM KCl in HBS-LB (isotonically balanced by reducing the [NaCl]) to determine the total number of viable Fluo-4 AM loaded TGNs in the image field. The intensities of recorded fluorescence signals (F) were analysed offline. Regions of interest (ROIs) were applied to individual TGN somata, and F was measured for each ROI in every frame of the video recordings using the average pixel intensity tool in Image J (version 1.53e, National Institutes of Health, USA). Values were exported to Microsoft Excel^®^ (Office 365, Microsoft Corporation, St Redmond, WA, USA) for further analysis. Measurements for time points recorded during the first 5 min were averaged to determine the initial fluorescence intensity (F_0_) and the s.d. in this baseline signal over this period. Changes in F relative to F_0_ were calculated for every time point using the formula (F − F_0_)/F_0_. ROIs that exhibited an increase of fluorescence such that (F − F_0_)/F_0_ was greater than (F_0_ + 10 × s.d.)/F_0_ were considered to be responders. Mean values and s.e.m. were determined for each time point from all responders and plotted against elapsed time using GraphPad Prism 9 (GraphPad Software, San Diego, CA, USA). The AUC of traces generated for each [AITC] were determined using the tool available in the latter program.

### 4.9. Data Analysis

Data were calculated in MS Excel and graphs were generated in GraphPad Prism 9; each point or bar represents a mean value, and all error bars signify s.e.m. or S.E. as indicated in Figure legends. AITC dose-dependent relationships were fitted using the formula: Y = min. + (max. − min.)/(1 + 10^((LogEC_50_-X)*Hill slope)). Welch’s unpaired t-test, one- or two-way analysis of variance (ANOVA) with posthoc tests for comparisons between individual points was used to evaluate the significance of changes. Statistical significance was attributed to differences between groups when *p* < 0.05. Asterisks or hashtags indicate *p* values; **** or ####, *p* < 0.0001; ***, *p* < 0.001; ** or ##, *p* < 0.01; *, *p* < 0.05.

## Data Availability

Data are contained within the article.
